# Advances in Rehabilitation Science and Practice (AdvRSP) Editorial: A Journal Showcasing Exciting Developments in the Field of Rehabilitation in Medical Conditions

**DOI:** 10.1177/11795727221139517

**Published:** 2022-11-29

**Authors:** Manoj Sivan

**Affiliations:** Academic Department of Rehabilitation Medicine, University of Leeds, Leeds, UK; President, British Society of Physical & Rehabilitation Medicine (BSPRM); Editor-in-Chief *Advances in Rehabilitation Science and Practice*

We at SAGE are excited to announce to our readers and authors several ambitious changes to Rehabilitation Process and Outcome. The title of the journal will be changing to *Advances in Rehabilitation Science and Practice*.

The journal aims to capture cutting-edge research and developments in the clinical practice of rehabilitation in medical conditions affecting adults and children. We will publish innovations from the field of neurorehabilitation, musculoskeletal conditions, sports sciences, trauma, chronic pain, cardiopulmonary rehabilitation, stroke, rehabilitation technology, outcome measurement and other areas related to rehabilitation.

Apart from researchers in the field of rehabilitation, this journal is also aimed at a wide variety of clinical specialists in the field – physicians specialising in Physical and Rehabilitation Medicine (also known as Rehabilitation Medicine or PM&R), physiotherapists, occupational therapists, nurses, psychologists, speech and language therapists, dieticians, support workers and other allied healthcare professionals working in the area of rehabilitation for chronic conditions. The journal is also likely to attract articles from related fields like Neurosciences, Orthopaedics and Trauma, Palliative care, Geriatrics, Cardiology and Respiratory Medicine.

*Advances in Rehabilitation Science and Practice* will continue to retain its focus on processes and outcomes in rehabilitation but wishes to expand its remit to basic sciences, clinical trials, qualitative research, clinical interventions, patient and public involvement, as well as health services and policy research. Apart from our continued emphasis on original research, systematic review and clinical trials, the journal aims to have the following special features

- Special Issues focusing on a particular area of interest (for example, *Long COVID: Mechanisms, Treatments and Outcomes* is currently open for submissions)
- Practice Pointers to capture the concise and logical approach to the management of common symptoms or conditions seen in rehabilitation settings
- Summary of clinical guidelines written by experts in the field
- Invited editorials, opinion articles or book reviews

The journal will strive to complete the initial review within 6 weeks of submission, which will involve at least 2 independent peer-reviewers and publish accepted articles within 3 weeks of acceptance. Accepted articles might be edited professionally by our editorial and production team for language and consistency of format to ensure consistent high-quality publications. As a SAGE pure gold open access journal, the journal is supported by the payment of an article processing charge (APC) by the author, institution or research funder of the accepted manuscript.

We are delighted to welcome the newest members of the Senior Editorial Team, who have joined us to manage the expanded scope of the journal, facilitate a robust peer-review process, manage special issue editions and shape our strategic vision. They come from various rehabilitation disciplines and have an impressive track record in basic science research and clinical practice. Some of them have championed translational research and are keen to see *Advances in Rehabilitation Science and Practice* publish articles with a direct impact on improving care for patients with chronic conditions and and make a difference to their function and quality of life.

We acknowledge the presence of other journals in this space doing the same but truly believe we have the ambition and the right team to build our reputation from here and become one of the most reputable journals in the field. This is an exciting phase of our growth and our vision to see *Advances in Rehabilitation Science and Practice* become the go-to journal in the fascinating area of rehabilitation research and clinical practice. We welcome your suggestions to make the journal highly impactful and improve the lives of individuals with rehabilitation needs, to whom this journal is dedicated.

